# Glazes induced degradation of tea catechins

**DOI:** 10.1038/s41598-023-37480-8

**Published:** 2023-06-28

**Authors:** Yunzi Xin, Sota Shido, Kunihiko Kato, Takashi Shirai

**Affiliations:** 1 Advanced Ceramics Research Center, Nagoya Institute of Technology, Nagoya, 466-8555 Japan; 2 Department of Life Science and Applied Chemistry, Graduate School of Engineering, Nagoya Institute of Technology, Nagoya, 466-8555 Japan

**Keywords:** Engineering, Materials science

## Abstract

In present work, the degradation behavior of tea catechins on various commercial glazes was elucidated for the first time. Four kinds of Japanese typical commercial glaze powders (Oribe /Namako/Irabo /Toumei) based on Fe/Co /Cu /Ti oxides were utilized and deposited on ceramic tiles. Tea solution extracted from green tea leaves at 80 °C and then utilized for the examination of degradation behavior with glazes to meet a nearly identical condition in human daily tea drinking with ceramicwares. It was found that the degradation of tea catechins significantly dependent on the chemical structure of glazes, that is: Fe/Cu/Co oxides contained glazes can promote the degradation of epigallocatechin, epicatechin, epigallocatechin gallate and epicatechin gallate, while Ti oxide contained glaze stimulated the degradation of epigallocatechin gallate selectively. Coloring pigments were produced in degraded tea solutions, whose color shows glaze dependent property. We presume that these color pigments can be assigned as oxytheotannin, especially theaflavin and its oxides as well as thearubigins, that produced through the polymerization of intermediate free radical catechin and/or the ortho-quinone generated by catalytic effect of glaze oxides worked as Lewis’s acids. The specific function of glazes on degradation of catechins discovered here not only provides principal information for design and development of functional materials but also bring new impacts on daily tea drinking and long-term human health-related issues.

## Introduction

Tea, with a long history date back to around 2700 B.C. has become one of the most popular drinks in human’s daily life due to the existence of natural flavonoids such as catechins which have been reported as protective agents against cardiovascular disease and cancer^1–5^. The degradation of different tea catechins has been studied with respects to the effects of pH and temperature in lab level. Konatsu et. al. reported that reaction of epicatechin (EC) was accelerated when pH is higher than 6.0 while inhibited under pH lower than 5.0 with isomerization reaction occurred in acidic media^6^. The reaction of tea catechins including epicatechin (EC), epicatechin gallate (ECg), epigallocatechin (EGC) and epigallocatechin gallate (EGCg) fitted an apparent first order reaction kinetics at temperature below 95 °C. Chen’s group studied the degradation of variable green tea catechins under higher temperatures up to 98 ℃and 120 °C and they suggested that epimerization of EGCg to gallocatechin gallate (GCg) was observed^7^. In addition, Liang’s group reported the degradation of catechins in alkaline solutions under various lights and it was demonstrated that free radical species can be generated followed with polymerization via a photoreaction^8^.

Although the canned and bottled tea is well developed, lots of people prefer to enjoy tea drinks made by utilizing traditional ceramic tea pots and cups in the pointing view of pursuing better flavor and aesthetics of tea culture. It has also been reported that the amount of total catechins in tea drinks directly extracted from tea leaves is much more than that of canned and bottled tea^7,9^. Ceramic tea pots and cups are conventionally fabricated by coating ceramic-glass glaze on green tiles followed with firing process. The coated glazes play important roles not only in the sealing of porous surface on ceramic tile, but also enable a great variety of color decorations induced by addition of altered metal oxide species. Different colors can be then achieved through the thermal process that involves structure-controlled crystallization of different metal oxides-based phases^10^. The specific function of glaze including the photocatalytic activity and the antibacterial ability have been reported previously^11,12^. However, the effect of glaze on the chemical structure change of tea catechins has not been studied, which cannot be ignored in the real case of drinking tea via ceramicwares. Here in this study, we investigated the degradation of catechins in green tea through commercial glaze surface as Oribe/Namako/Irabo/Toumei which consist of different metal oxides of Cu/Co/Fe/Ti. Interestingly, it was found that the degradation behavior of EC, ECg, EGC and EGCg in green tea significantly dependents on the chemical structure of ceramic glazes. The specific function of glazes on degradation of catechins discovered here not only provides principal information on material science but also bring new impacts on daily tea drinking and long-term human health-related issues.

## Results and discussion

**Figure 1:**
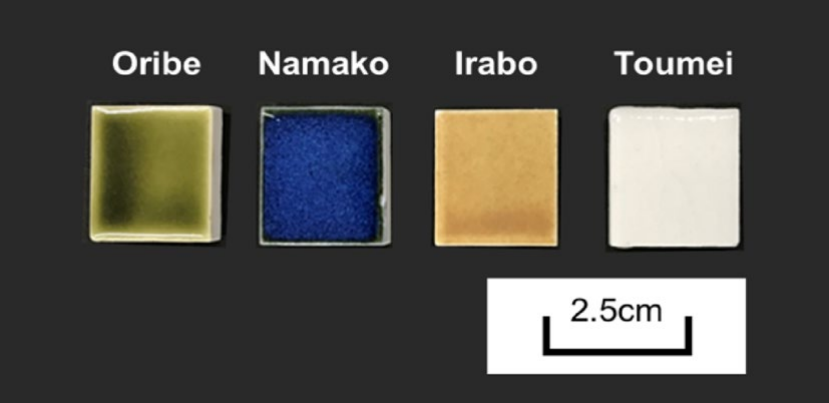
Appearance of glaze sample pieces after sintering at 1250 °C.

The chemical compositions of metal elements in glazes analyzed by XRF are summarized in Table 1. As the results demonstrated, glaze samples consist of variable metal oxides originated from the mineral natures. The existence of Si/Al/K/Na/Ca corresponding to SiO_2_, Al_2_O_3_, K_2_O, Na_2_O and CaO in all glaze sample can be attributed to the feldspar, which is the main natural mineral source in glaze manufacturing. Compare with results of tile substrate, it can be concluded that the major metal specie involved in Oribe glaze is Cu, while Namako glaze includes mainly Co and Ti. Fe and Ti are the main metal sources in Irabo and Toumei glazes, respectively. The metal elements detected in XRF also show good agreement with the color appearance of sintered sample pieces as shown in Fig. 1: that is, green, blue, orange, and transparent coloring corresponding to Cu, Co, Fe and Ti, respectively.

**Table 1:** Element composition of tile and variable glaze sample pieces after vacuum drying.

Composition element	Si	Al	P	Zr	K	Mg	Na	Ca	Fe	Ti	Cu	Co	Ba	Impurity
*Tile*	66.3	23.1	2.7	2.2	2.1	1.7	1.1	0.3	0.3	0.2	–	–	–	–
*Oribe*	57.6	15.1	1.1	2.9	4.9	–	–	11.2	0.7	0.2	**3.3**	–	–	0
*Namako*	57.8	10.9	–	1.7	3.1	1.3	–	9.8	0.8	**4.6**	–	**2.5**	5	2.5
*Irabo*	42.6	19.2	2.1	2	2.8	2.3	1.4	21.9	**3.5**	–	–	–	–	2.2
*Toumei*	69.1	12.5	–	0.8	4.6	–	1.3	8.8	–	**2.8**	–	–	–	0.1

**Figure 2:**
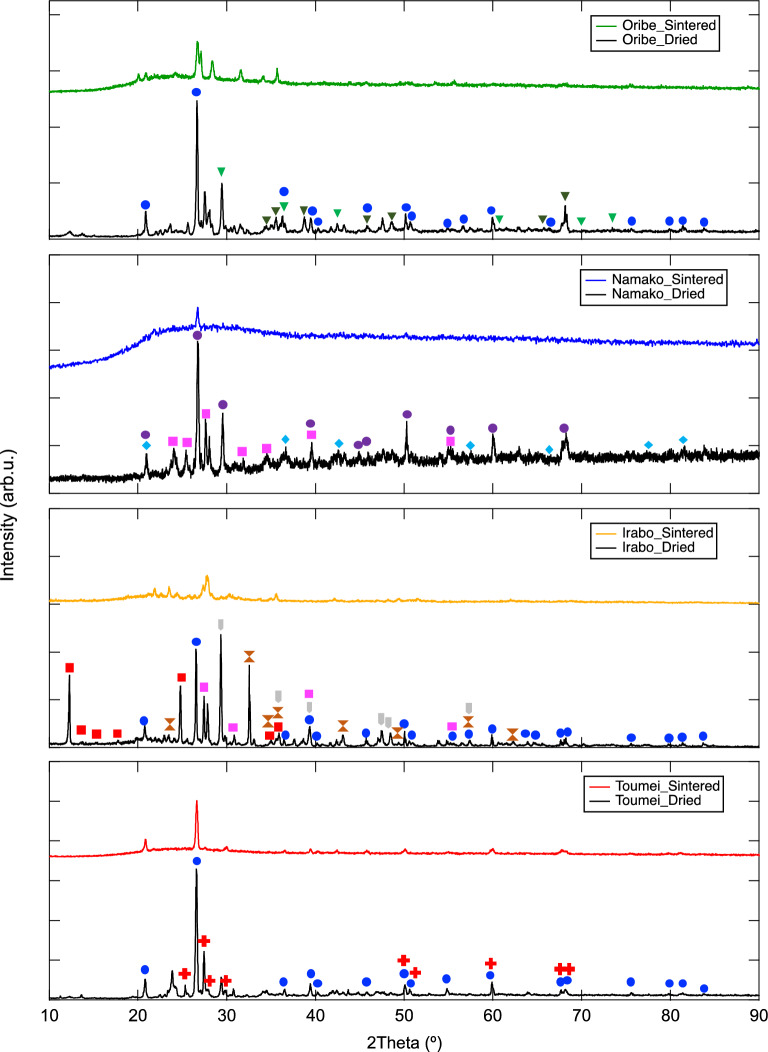
PXRD spectra of glaze sample pieces after drying and sintering at 1250 °C (blue circle: quartz, purple circle: coesite, pink square: proxane-ideal, red square: antigorite; light-green triangle: Cu_2_O, dark-green triangle: CuO, water-blue rhombus: CoO_2_, brown spindle: Fe_2_O_3_, bright-grey rectangle: CaCO_3_, red cross: anatase TiO_2_).

The PXRD results of glaze sample pieces before and after sintering at 1250 °C are illustrated in Fig. 2, respectively. In the case of before sintering, crystal phases of SiO_2_ component were observed in all glaze pieces. Quartz phase is the main component in Oribe, Irabo and Toumei while coesite phase appears in Namako. In addition, pyroxene (MgO_3_Si) was confirmed in Namako and Irabo, which meets good agreement with confirmation of Mg element in XRF results demonstrated in Table 1. With existence of Fe and Ca, antiqorite ((Mg, Fe)_3_Si_2_O_5_(OH)_4_) and CaCO_3_ were also observed in Irabo sample. For the main metal components probably gave coloring appearance of sample pieces, Cu_2_O/CuO, CoO_2_, Fe_2_O_3_ and anatase TiO_2_ were confirmed in Oribe, Namako, Irabo and Toumei, respectively. With glass phase formed on glaze surface after sintering process, the existence of metal oxides except Si-based component became difficult to be confirmed in PXRD patterns. Regards to the crystal phases of metal oxides confirmed in PXRD before sintering and specific color appearance observed in sample pieces shown in Fig. 1, we conclude that the trace amount of metal oxides might exist in sintered glazes. In following parts, the degradation behavior of tea on different glazes and the influence of metal oxides on structure change of catechins will be discussed, whose investigation reflects the real situation during tea drinking on ceramicwares.

**Figure 3:**
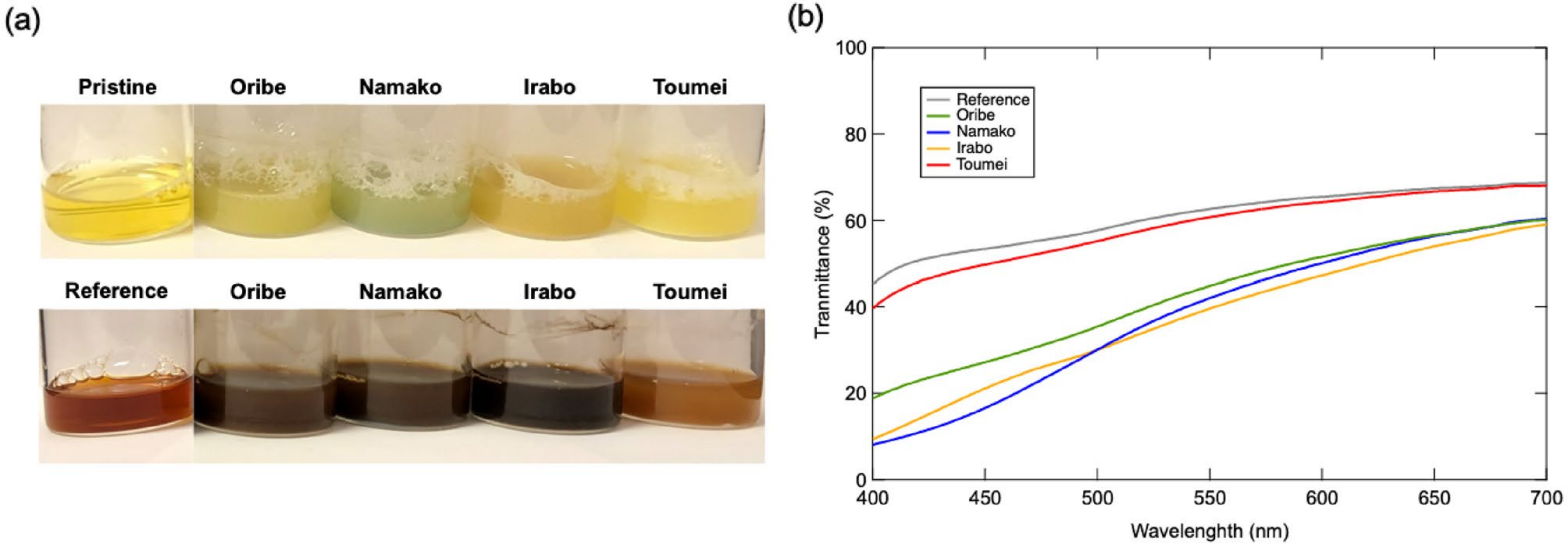
(**a**) Photograph of pristine tea solution and 6 h-degraded tea solution with addition of Oribe, Namako, Irabo and Toumei glaze powders, (**b**) corresponding UV–vis spectra after degradation.

**Figure 4:**
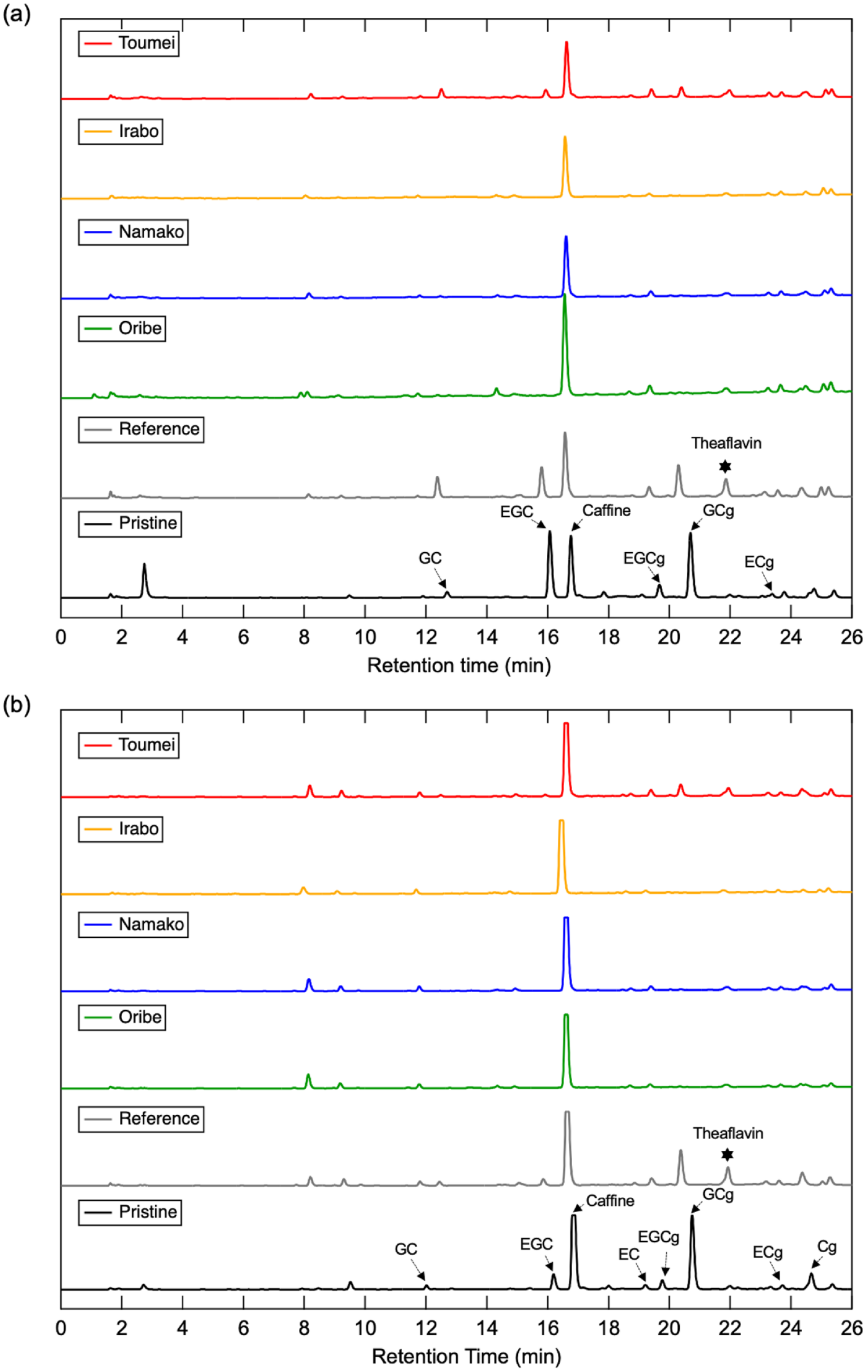
HPLC spectra of tea solutions before and after 6 h degradation with and without addition of glaze powders detected at (**a**) 242 nm and (**b**) 272 nm.

**Figure 5:**
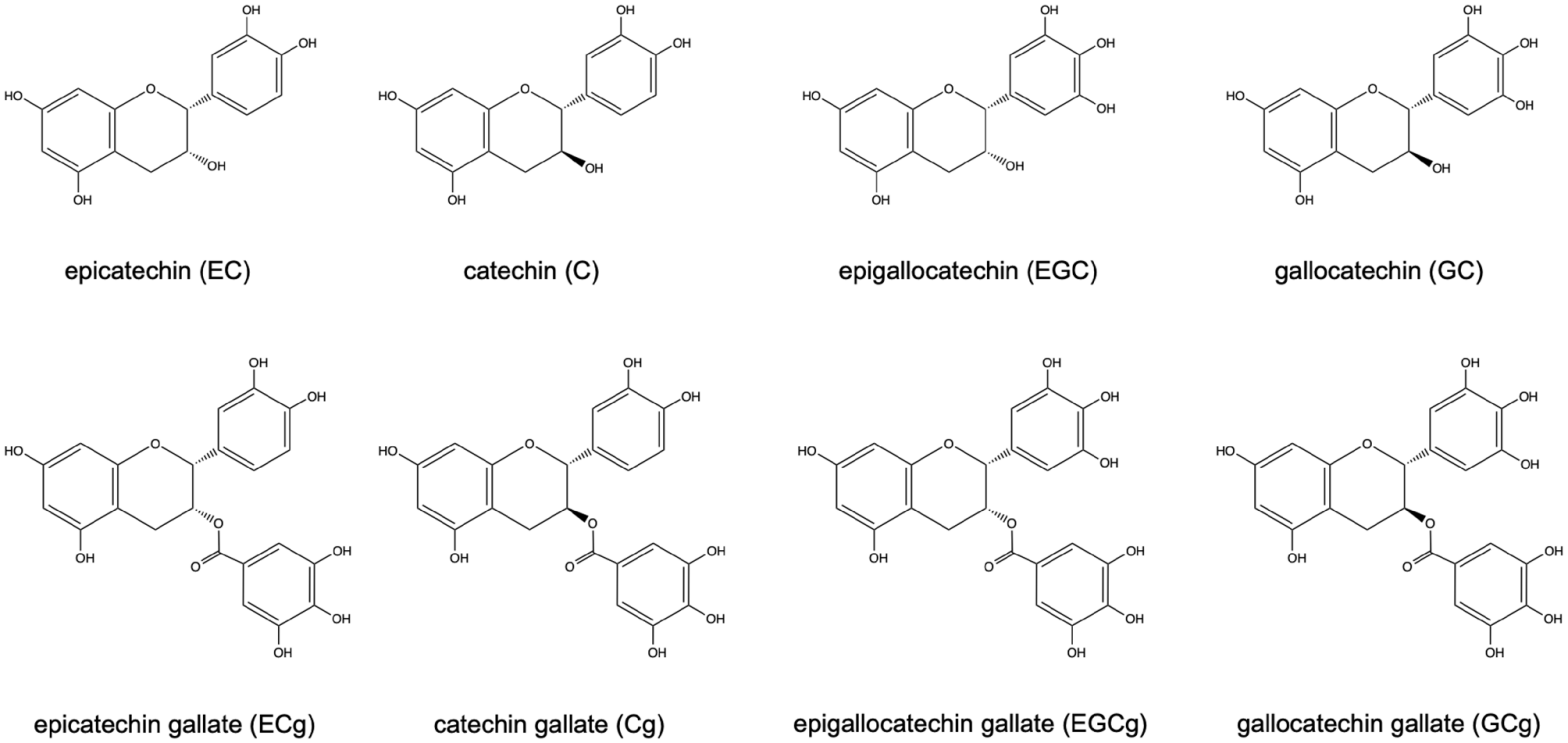
Chemical structure of catechin molecules (topside: catechins, downside: catechin gallates).

**Figure 6:**
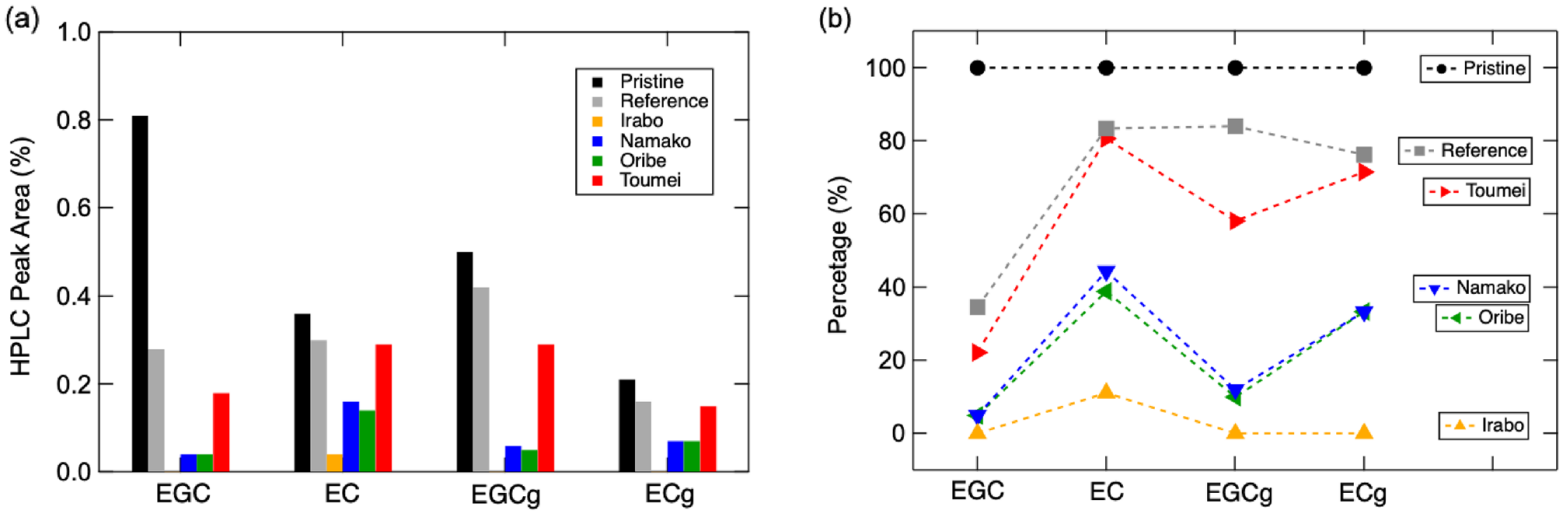
(**a**) Integrated peak area of each catechin observed in HPLC spectra and (**b**) amount of each catechin after degraded with different glazes by setting amount in pristine tea solution as 100%.

Figure 3a reveals the photographs of pristine tea solution and after degradation for 6 h with/without existence of glaze powders. It is obvious that pristine tea shows clear bright yellow color and self-degraded into yellowish brown color after 6 h, while tea solutions degraded into dark black/brown color with existence of different glaze samples. In addition, the degree of color change depends significantly on the type of glazes that consist of different metal oxides. Brownish black was observed in degraded tea solution of Oribe (Cu), Namako (Co) and Irabo (Fe) glazes, while dark brown solution observed in Toumei (Ti) glaze. For further investigation of the above color change phenomenon, visible light transmittance of each degraded solution is evaluated via in UV–vis spectroscopy. As the results shown in Fig. 3b, tea solution degraded without existence of glaze exhibits the highest transmittance in 300 ~ 700 nm region, while the transmittance of tea solutions degraded with glaze decreases with reduced transmittance in order of Toumei < Oribe < Irabo <  = Namako. To clarify the reason for color changing of tea solution after degradation, the catechin components in tea solutions are investigated via HPLC measurement. The HPLC spectra detected at different wavelengths of 242 nm and 272 nm are demonstrated in Fig. 4. Peaks of EGC, EC, EGCg, ECg, GC (gallocatechin), GCg (gallocatechin gallate), and Cg (catechin gallate) appear with retention times as main catechin components are observed, where EC, ECg and Cg appears more obviously in spectra of 242 nm than 282 nm. The detailed chemical structure of these catechins and their gallate molecules are represented in Fig. 5. C and GC are isomers of EC and EGC. EGC exhibits familiar structure with EC, where pyrogallol group in EGC is replaced by a catechol group in EC. In the case of tea solutions degraded with different glaze, the amounts of all catechins including GC, EGC, EGCg, EC, GCg, ECg, ECg and Cg reduces significantly than the reference tea solution that degraded without addition of glaze. For better understanding, the integrated peak area of each catechin is summarized in Fig. 6a. To set the amount of each catechin in pristine as 100%, the corresponding amount after degradation with existence of different glazes is illustrated in Fig. 6b. The results suggest that the amount of catechins such as EGC, EC, EGCg and EGC were reduced more significantly with existence of Irabo, Oribe and Namako glazes, while Toumei glaze seems show fewer effects on degradation of tea solution with selected response to EGCg only. Meanwhile, it can be also demonstrated that EGC and EGCg show predominated degradation with glazes than EC and ECg. To compare with tea solution that self-degraded, it can be concluded that tea catechin degraded with the glaze powders show a distinguished unique behavior.

In addition, a new peak appear around retention time of 22 min is observed in degraded tea solutions (marked by star symbol in HPLC spectra), whose peak intensity is higher in reference tea solution than glaze-included ones. The chemical structure was identified as theaflavin formed via reaction between catechin (C)/EC and EGC/GC by LC-TOF–MS analysis and the result is ascribed in Fig. S1 in Electronic Support Information (ESI). It has been previously reported that the EGC, EC, EGCg and ECg are degraded into theaflavins through oxidation and polymerization companying with color changes from colorless into black^13^. Thus, the color changing in degraded tea solutions (Fig. 3) and peak reduction of catechins in HPLC spectra (Fig. 6) might be partially attributed to the formation of theaflavin. However, due to the fact that the peak intensity of theaflavin comes out to be much higher in reference tea solution than the ones with Toumei as well as other glazes, it can be considered that a distinguished degradation mechanism with generation of color pigments was also established in glaze added case.

**Figure 7:**
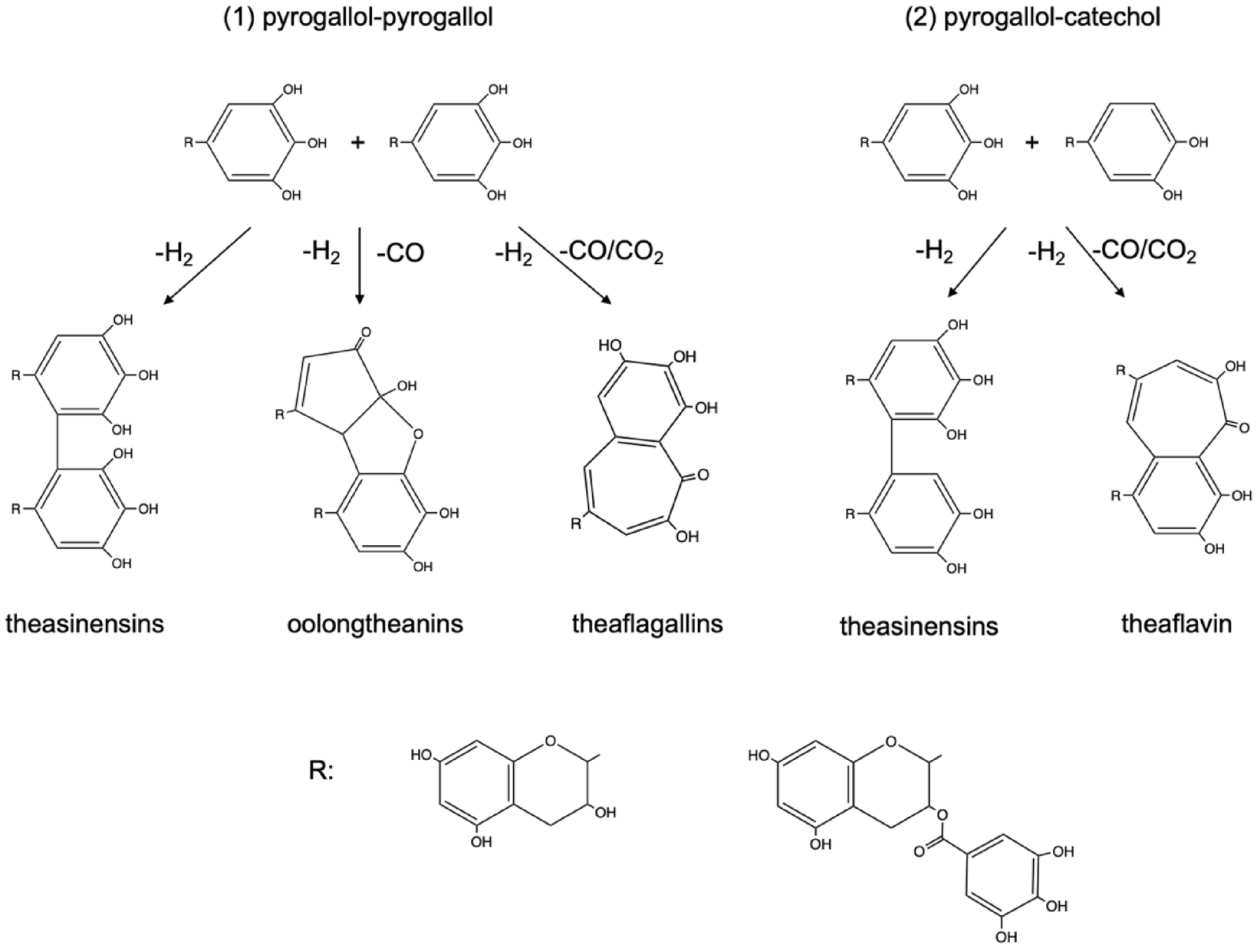
Two possible oxidation strategies towards generation of dimeric oxytheotannin.

Oxytheotannin, known as color pigment, represents dimeric, oligomeric and/or polymeric compounds produced through oxidation of polyphenol such as catechin molecules has been demonstrated previously. Oxytheotannin can be divided into two species including theaflavin that shows reddish-orange color, and thearubigins which exhibit brownish color, respectively^14^. These color pigments are also known as the main components in semi-fermented oolong tea as well as fermented black tea. Although theaflavin and thearubigins were firstly discovered in 1960s, the exact chemical structure of oligomeric and polymeric oxytheotannins remains ambiguous due to the complex oxidation mechanism in the past 60 years. As an example, for dimeric oxytheotannins, five species products (as shown in Fig. 7) can be generated through two different oxidation strategies have been reported by Hashimoto et al^15^, that is: (1) pyrogallol-pyrogallol dimerization will produce theasinensins, oolongtheanins and theaflagallins by release H_2_, 2H_2_/CO and 2H_2_/CO/CO_2_, respectively, and (2) theasinensins and theaflavin will be obtained through dimerization of pyrogallol-catechol with extrusion of H_2_ and 2H_2_/CO, respectively. It is worth noting that these dimeric products, for example theaflavin, will also oxides further via polymerization and condensation^16^. As example, S. Sang^17^ reported that theaflavin can further react with catechin to form di- or tri-benzotropolone molecules which also cause color changes. In addition, Kusano suggested that theacoumarin will also be generated through deep oxidation of theaflavin^18^. Thus, the less amount of theaflavin observed in tea solution degraded by Irabo, Oribe and Namako glaze powders but darker color appearance can be attributed to two possible reasons, that is: (1) unknown thearubigins with complex oligomeric and polymeric structures were produced, (2) generation of theaflavin followed by further oxidation occurs. And due to the fact that the above oxidation reactions can be influence by both metal ions and oxides, we discuss the roles of metal ions released from glaze powders that dissolved in tea solution, and metal oxide exposed on glaze surface in present study as follows.

Regards to the fact that small amount (less than 10 ppm for each metal species) of metal ions diluted from glazes were detected through ICP-OES analysis, reaction between metal ions and catechin/theaflavin molecules should be counted. We discuss these reactions point-to-point as follows with respects to altered strategies: (i) it has been reported that diluted metal ions inhibit the formation of theaflavin through degradation of catechins, whose mechanism can be attributed to the reduced acid dissociation constants of EC, EGC, ECg and EGCg with existence of Cu^2+^, Fe^2+^/Fe^3+^.^19^ These results help to explain the reduction of catechins species but trace appearance of theaflavin in HPLC spectra (Fig. 4&5) for the degradation with glaze powders. (ii) it has been also reported that diluted metal ions as Cu^2+^, Fe^2+^/Fe^3+^ and Co^2+^ react with catechol group and form into metal complex^20,21^, whose process may contribute to the reduced amount of GC, EGC, EGCg, EC, ECg, and ECg as observed from HPLC results. As additional experiment, the degradation of tea catechins with metal ions such as Fe^2+^ and Cu^2+^ was also conducted, following by HPLC analysis of degraded solutions. As the results shown in Fig. S2, it suggests that there is no significant difference between Fe^2+^ and Cu^2+^ added samples with reference one. Thus, it demonstrates that the reaction between metal ions and catechins can be ignored in current case. In addition, it has been demonstrated that such metal complex exhibits characteristic absorption band around 312 nm^18^. By monitoring the absorption spectra in UV region (as shown in Fig. S3), it suggests that the there is no obvious peak appear near 312 nm but increased absorption in entire UV region, especially around 260 ~ 280 nm. It has been reported that the characteristic absorption around 270 nm can be attributed to the typical π − π* transition of catechin molecules and related oxides^22^. (iii) we also considered the possibility of secondary reaction between metal ions and pre-generated theaflavin. O’Coinseananim et al. reported that characteristic FTIR peaks of C = O will be altered when theaflavin react with Al^3+^ ion^23^. To clarify the chemical structure changing of catechins and theaflavin, the FTIR spectra of products that degraded by glaze powders were characterized via ATR attachment. The observed spectra are displayed as Fig. S4. Although the peak of C = O near 1600 cm^−1^ changed between self-degradation and that degraded with glaze powders, the rare differences for different glaze species demonstrates that such secondary reaction between metal ions with theaflavin might be not the principal reason for color change after degradation with glaze powders.

**Scheme 1.**
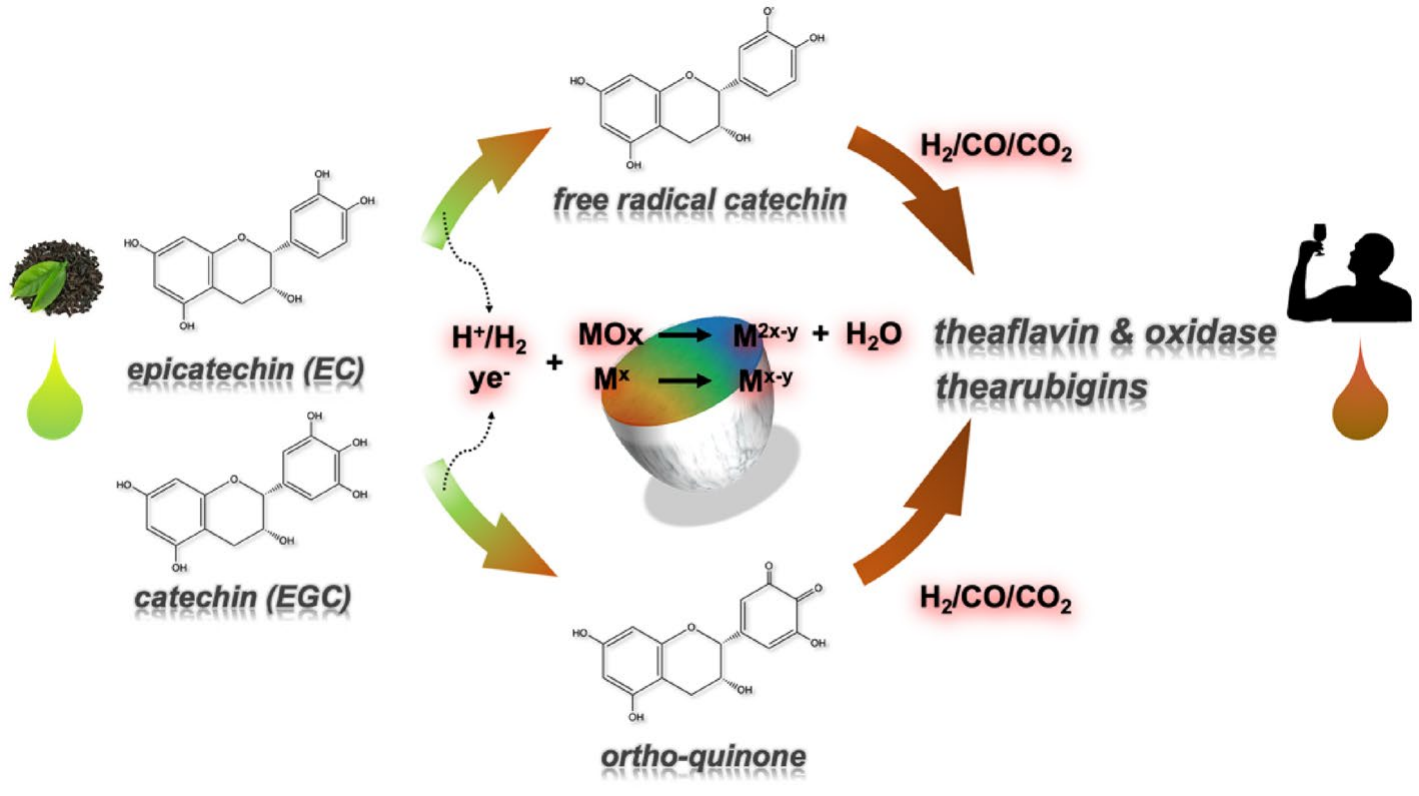
Presumed mechanism for glaze-induced degradation of tea catechin through two altered routes.

For the effect of metal oxides, Wang’s group reported that short-range order Mn-, Fe- and Al-oxides can catalyze the polymerization of catechin^24^. They demonstrate that these metal oxides containing metal elements with high oxidation numberers can act as Lewis’s acid and accepting electrons transferred from catechin molecule. Thus, we propose such as Cu-, Co-, Fe- and Ti-oxides in glaze powders can act as Lewis’s acid catalyst and promote the oxidation of catechin molecules as well as further reaction of theaflavin. The presumed mechanism is discribed as Scheme 1. Whatever the hypothesis that oxidation of catechin through one electron by giving free radical catechins, nor by two electrons oxidation to generate otho-quinone^14^, the generated electrons and protons can react with metal with high oxidation numbers. Although the exact chemical structure and composition of oxidation product generated via degradation was difficult to be characterized, the results especially different degradation behavior of tea catechins on commercial glaze surface reveal important insights of heterogenous reaction through metal oxides surfaces/interface.

## Conclusions

In conclusion, the degradation of tea catechins on four kinds of commercial glaze materials as Oribe/Namako/Irabo/Toumei which containing metal oxides of Fe/Co/Cu/Ti elements, respectively, were investigated to look inside the reaction between tea and ceramicware occurs on glaze surface during tea drinking in human daily life. The chemical structure of glazes in fabricated sample pieces was confirmed by XRF and PXRD. Tea solution extracted from green tea leaves at 80 °C and then utilized for the examination of degradation behavior with glazes. As a result, the degradation behavior of typical tea catechins such as epigallocatechin, epicatechin, epigallocatechin gallate and epicatechin gallate significantly depends on the chemical structure of glazes. Regards to the HPLC result, it illustrates that Fe/Cu/Co oxides contained glazes can promote the degradation of epigallocatechin, epicatechin, epigallocatechin gallate and epicatechin gallate, while Ti oxide contained glaze promotes the degradation of epigallocatechin gallate selectively. Coloring pigments were produced in degraded tea solutions, whose color shows glaze dependent property. Two possible degradation mechanism of tea catechins on glaze surface are presumed as follows: (1) unknown thearubigins with complex oligomeric and polymeric structures were produced, (2) generation of theaflavin followed by further oxidation occurs. During the degradation process, Cu-, Co-, Fe- and Ti-oxides in glaze powders can act as Lewis’s acid catalyst and promote the oxidation of catechin molecules as well as further reaction of theaflavin. Although the exact chemical structure of the final products of oxytheotanni couldn’t be identified due to the complicated polymerization mechanism of nature tea catechins, the new insights elucidated in present work provides direct evidence that ceramicware does change the flavor of tea. We believe these contents also provides important information on design and development of functional ceramic material, whose output also correlated to the long-term human health-related issues. In near future, we will pay more efforts on clarification of the chemical structure and compositions of the oxytheotanni products via sufficient techniques.

## Methods

### Preparation of glaze sample pieces

**Scheme 2:**
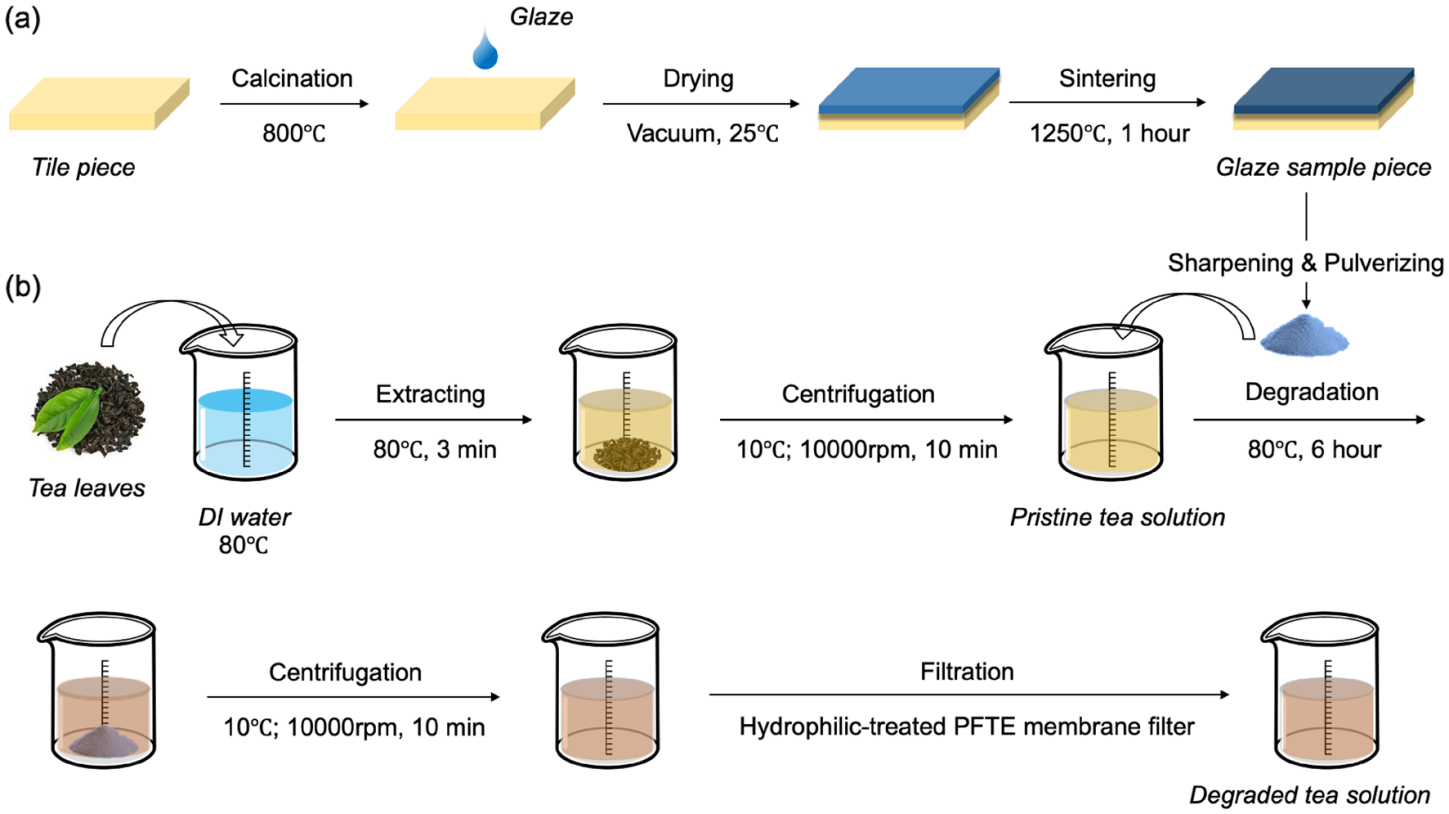
(**a**) Preparation process for glaze sample pieces and (**b**) experimental procedure for tea degradation testing.

Four kinds of typical domestic commercial glazes named as Oribe, Namako, Irabo and Toumei (Mizuno Chemical Co., Ltd.) were chosen as the main materials in this work. Sintered glaze sample pieces were fabricated by following process. Firstly, tile pieces were calcinated under 800 °C under air atmosphere. Secondly, different glazes were dip-casted on tiles followed with vacuum drying under 25 °C for 12 h. Sintering based on the most utilized conventional process was then conducted on dried samples at 1250 °C for 1 h. The above process is illustrated as Scheme 2a.

### Characterizations of glaze samples

Identification and quantitative analysis of the chemical elements contained on the surface of prepared glaze sample pieces were performed on an energy dispersive X-ray fluorescence analyzer (XRF: EDX-800H, Shimadzu Corp.). The crystal structure in glaze samples was analyzed using a powder X-ray diffractometer (PXRD: Ultima IV, Rigaku Corp.) equipped by a Cu Kα beam at operating current/voltage of 40 mA/40 mV. The chemical state and radical generation of metals in glazes sample was characterized by electron spin resonance (ESR) spectroscopy (JES-FA200, JEOL Ltd).

### Degradation of tea solution on glazes

To increase the interaction between tea and glazes, glaze powders are prepared by sharpening and pulverizing of sintered glaze sample piece surface. 1.00 g of tea leaves of a commercial Japanese green tea (Sencha, Jun, Myokoen Co. Ltd.) are weighed and tea solution was extracted with 25 mL of ion-exchanged water at 80 °C for 3 min. The leachate was centrifuged at 10,000 rpm at 10 °C for 10 min on a centrifugal equipment (3-16KL, SIGMA Corp.), and the supernatant was used as the pristine tea solution for catechin degradation study. For the reaction between tea catechin and glaze powders, 0.15 g of glaze powders and 15 mL of pristine tea solution was mixed in a screw vial, and reaction was processed for 6 h in a shaking bath maintained at 80 °C for 6 h. The glaze powder was removed by high-speed centrifugation (10,000 rpm, 10 min) and filtration by hydrophilic-treated PTFE membrane filter with pore size of 200 nm. The detailed experimental procedure is illustrated as Scheme 2b.

### Characterization of tea solution before and after degradation

The degradation of catechins in tea was studied in details with assistance of high-performance liquid chromatography (HPLC: pump-PU-4180/detector-UV-4075, JASCO Corp.) by applying reverse phase chromatography with an ODS column was utilized. Standard catechins of EGC, EGCg, EC, ECg, GC and GCg, (Funakoshi Co., Ltd) were used to confirm the catechins type appear in HPLC spectra. The color changing in different degraded solution was confirmed by transmittance measurement on an UV–visible spectroscopy (UV–vis: V-750, JASCO Corp.). The chemical component besides the standard catechins was identified by a LC mass spectroscopy with time-of-flight detector (LC-TOF–MS, Waters Corp.). In addition, the metal ions diluted from glaze through degradation were confirmed by inductivity coupled plasma optical emission spectrometer (ICP-OES: ICPE-9820, Shimadzu Corp.)

## Supplementary Information

Supplementary Information.

## Data Availability

The datasets used and/or analyzed during the current study available from the corresponding author on reasonable request.
