# Author Correction: Glazes induced degradation of tea catechins

**DOI:** 10.1038/s41598-023-39072-y

**Published:** 2023-07-25

**Authors:** Yunzi Xin, Sota Shido, Kunihiko Kato, Takashi Shirai

**Affiliations:** 1 Advanced Ceramics Research Center, Nagoya Institute of Technology, Nagoya, 466-8555 Japan; 2 Department of Life Science and Applied Chemistry, Graduate School of Engineering, Nagoya Institute of Technology, Nagoya, 466-8555 Japan

Correction to: *Scientific Reports* https://doi.org/10.1038/s41598-023-37480-8, published online 28 June 2023

The original PDF version of this Article contained a repeated error where the oxide “Co” was incorrectly given as “Mg” in the Abstract and Conclusions.

The original Article has been corrected.

