# Supplementary material for: Glazes induced degradation of tea catechins
Source: Sci Rep. 2023 Jun 28;13:10507. doi: 10.1038/s41598-023-37480-8 (PMC10307877; doi:10.1038/s41598-023-37480-8)
Supplement: Supplementary file 1 — Supplementary Information. [file 41598_2023_37480_MOESM1_ESM.docx]

　　Supplementary Information

**Glazes Induced Degradation of Tea Catechins**

Yunzi Xin,^1^ Sota Shido,^2^ Kunihiko Kato,^1^ Takashi Shirai^1,2,^*

[shirai@nitech.ac.jp](mailto:shirai@nitech.ac.jp)

*1. Advanced Ceramics Research Center 2. Department of Life Science and Applied Chemistry, Graduate School of Engineering, Nagoya Institute of Technology, Gokiso-cho, Showa-ku, Nagoya, Aichi, Japan 466-8555*


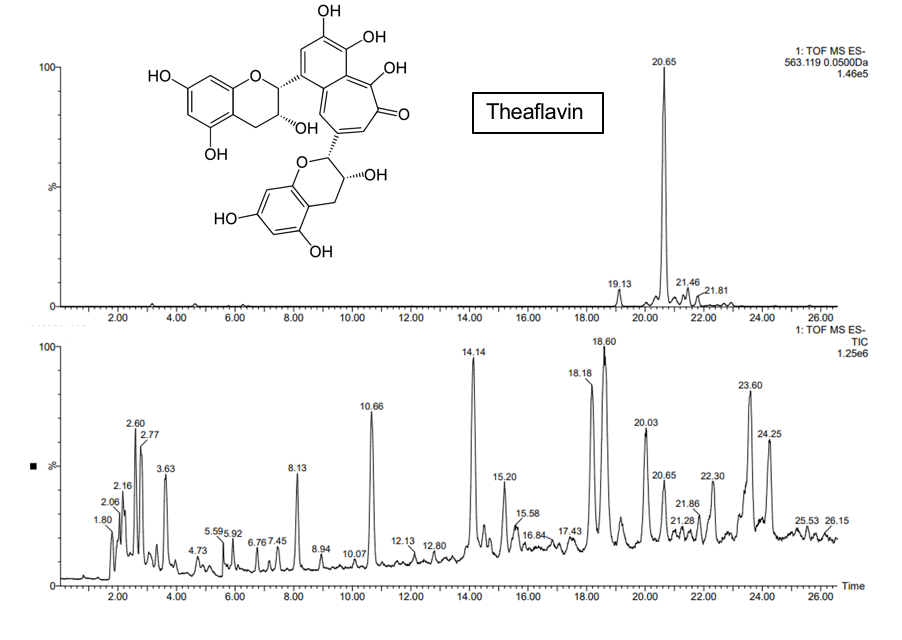


Figure S1. LC-TOF-MS spectra of reference tea solutions after 6-hours degradation without addition of glaze.

Figure S2. HPLC spectra of tea solutions after 6 hours degradation with addition of 20 ppm metal ions as Fe^2+^ and Cu^2+^, compare with the one without addition of metal ions.

Figure S3. Absorption spectra of degraded tea solutions.

Figure S4. FTIR spectra of tea solutions before and after 6 hours degradation with/without addition of glazes.
